# Mannan-Abeta_28 _conjugate prevents Abeta-plaque deposition, but increases microhemorrhages in the brains of vaccinated Tg2576 (APPsw) mice

**DOI:** 10.1186/1742-2094-5-42

**Published:** 2008-09-29

**Authors:** Irina Petrushina, Anahit Ghochikyan, Mikayel Mkrtichyan, Grigor Mamikonyan, Nina Movsesyan, Rodmehr Ajdari, Vitaly Vasilevko, Adrine Karapetyan, Andrew Lees, Michael G Agadjanyan, David H Cribbs

**Affiliations:** 1 The Institute for Brain Aging and Dementia, University of California Irvine, Irvine, CA 92697-4540, USA; 2 The Institute for Molecular Medicine, Department of Immunology, Huntington Beach, CA 92649, USA; 3 Biosynexus Incorporated, Gaithersburg, MD 20877, USA; 4 Professor of Neurology, Department of Neurology, The Institute for Brain Aging and Dementia, 1207 Gillespie NRF, University of California Irvine, Irvine, CA 92697-4540, USA

## Abstract

**Background:**

New pre-clinical trials in AD mouse models may help to develop novel immunogen-adjuvant configurations with the potential to avoid the adverse responses that occurred during the clinical trials with AN-1792 vaccine formulation. Recently, we have pursued an alternative immunization strategy that replaces QS21 the Th1 type adjuvant used in the AN-1792 clinical trial with a molecular adjuvant, mannan that can promote a Th2-polarized immune response through interactions with mannose-binding and CD35/CD21 receptors of the innate immune system. Previously we established that immunization of wild-type mice with mannan-Aβ_28 _conjugate promoted Th2-mediated humoral and cellular immune responses. In the current study, we tested the efficacy of this vaccine configuration in amyloid precursor protein (APP) transgenic mice (Tg2576).

**Methods:**

Mannan was purified, activated and chemically conjugated to Aβ_28 _peptide. Humoral immune responses induced by the immunization of mice with mannan-Aβ_28 _conjugate were analyzed using a standard ELISA. Aβ_42 _and Aβ_40 _amyloid burden, cerebral amyloid angiopathy (CAA), astrocytosis, and microgliosis in the brain of immunized and control mice were detected using immunohistochemistry. Additionally, cored plaques and cerebral vascular microhemorrhages in the brains of vaccinated mice were detected by standard histochemistry.

**Results:**

Immunizations with low doses of mannan-Aβ_28 _induced potent and long-lasting anti-Aβ humoral responses in Tg2576 mice. Even 11 months after the last injection, the immunized mice were still producing low levels of anti-Aβ antibodies, predominantly of the IgG1 isotype, indicative of a Th2 immune response. Vaccination with mannan-Aβ_28 _prevented Aβ plaque deposition, but unexpectedly increased the level of microhemorrhages in the brains of aged immunized mice compared to two groups of control animals of the same age either injected with molecular adjuvant fused with an irrelevant antigen, BSA (mannan-BSA) or non-immunized mice. Of note, mice immunized with mannan-Aβ_28 _showed a trend toward elevated levels of CAA in the neocortex and in the leptomeninges compared to that in mice of both control groups.

**Conclusion:**

Mannan conjugated to Aβ_28 _provided sufficient adjuvant activity to induce potent anti-Aβ antibodies in APP transgenic mice, which have been shown to be hyporesponsive to immunization with Aβ self-antigen. However, in old Tg2576 mice there were increased levels of cerebral microhemorrhages in mannan-Aβ_28 _immunized mice. This effect was likely unrelated to the anti-mannan antibodies induced by the immunoconjugate, because control mice immunized with mannan-BSA also induced antibodies specific to mannan, but did not have increased levels of cerebral microhemorrhages compared with non-immunized mice. Whether these anti-mannan antibodies increased the permeability of the blood brain barrier thus allowing elevated levels of anti-Aβ antibodies entry into cerebral perivascular or brain parenchymal spaces and contributed to the increased incidence of microhemorrhages remains to be investigated in the future studies.

## Background

The neuropathological features of Alzheimer's disease (AD) include neurofibrillary tangles (NFT), deposition of amyloid-β (Aβ) in senile plaques, and neuronal loss in affected brain regions [1]. The Aβ is cleaved from the amyloid precursor protein (APP) by the β- and γ-secretases [2-4], and is believed to play an important role in the onset and progression of AD [5,6]. Accordingly, many of the strategies currently being proposed as therapies for AD are aimed at reducing the level of Aβ in the brain, and/or preventing the assembly of this peptide into pathological forms. One potentially powerful strategy for reducing the level of Aβ in the brain is immunotherapy, in which antibodies specific for Aβ facilitate the clearance amyloid deposits and a reduction in other forms Aβ in the brain parenchyma. Both active and passive Aβ-immunotherapy significantly decrease amyloid deposits, neuritic dystrophy and gliosis in the brain, as well as improve behavioural measures in APP/Tg mice [7-12]. Based on these and other studies in several animal models, the AN-1792 clinical trial was initiated in patients with mild-to-moderate AD, however the trial was halted during the phase II portion of the trial when approximately 6% of the AD patients vaccinated with AN1792 developed meningoencephalitis and in some cases CAA associated haemorrhages. The cause of the encephalitis in these patients has not yet been determined, however speculation has focused on autoreactive T-cells and the proinflammatory Th1 type of adjuvant that was used in the clinical trial [13-16]. Of note, there is only one unconfirmed report about lymphocyte infiltration detected in the brains of wildtype mice immunized with Aβ_42 _formulated in FCA/FIA followed by injection with pertussis toxin [17]. In addition to T cell infiltration into the brain parenchyma, another participant in the clinical trial developed widespread cortical hemorrhages [16]. Microhemorrhages in the cerebral vasculature have also been observed in different strains of very old (21–26 month-old) APP/Tg mice injected weekly with high doses of anti-Aβ monoclonal antibodies, and the sites of microhemorrhage have been co-localized with cerebral vascular Aβ deposits [18-21]. Recently, it was reported that active immunizations of APP/Tg mice with fibrillar Aβ_42 _formulated in a strong conventional adjuvant, Freund's complete adjuvant system (FCA), could also increase microhemorrhages in the brains of vaccinated animals [22]. Collectively, these adverse events emphasized the need for further refinement of vaccines for AD in order to eliminate, or at least attenuate, the potential adverse events initiated by infiltration of autoreactive T cells and peripheral macrophages, as well as inflammation-induced cerebral vascular microhemorrhages.

Mannan and other polysaccharides are recognized by collectins, such as, Mannose-Binding Lectin (MBL) [23-25], as well as Mannose Binding Receptors (MBRs) expressed on dendritic cells, some endothelial cells and macrophages [26-29]. After binding to mannan, MBL initiates the complement pathway via an activation of the MASP-1 & MASP-2 proteases, and is antibody- and C1q-independent. Therefore, immunization of mice with Aβ peptide sequences conjugated to mannan should induce complement activation and C3d opsonization on the Aβ. Moreover, MBL, which also has opsonic function like complement C1q (homologue of MBL), binds to complement receptor type 1 (CR1/CD35) [30] and therefore stimulates phagocytosis of the conjugated antigen. Finally, T-cell dependent B-cell immune responses can be activated not only by simultaneous triggering of BCR and C' receptor type 2 (CD21), but also by simultaneous triggering of BCR and C' receptor type 1 (CD35) [31,32]. Importantly, different groups have already demonstrated enhancement of T-helper and B-cell immune responses against several mannosylated-antigens, including peptide-antigens [33-35]. In addition to the direct activation of C', Aβ-mannan conjugates will be recognized by MBRs found on dendritic cells, some endothelial cells and macrophages [26-28]. The macrophage mannose receptor (MMR) and DEC-205 found on dendritic and some endothelial cells bind multiple carbohydrates [36]. These receptors significantly enhance phagocytosis of antigens containing an appropriate carbohydrate, such as mannose residues. In addition, the receptors can also increase antigen presentation on macrophages and dendritic cells by 100- to 1,000-fold over antigens without carbohydrate residues. Thus, besides activating complement, Aβ conjugated to mannan will also promote phagocytosis and antigen presentation on macrophages and dendritic cells of the immune system, providing an added benefit from the conjugation of mannan to Aβ. Notably, under certain conditions, mannosylated antigens induced strong Th2-type anti-inflammatory responses (production of IL4/IL10 cytokines) with high levels of IgG1 antibodies [33,34,37,38].

Previously, we generated mannan-Aβ_28 _conjugate and demonstrated that immunization of wild-type mice with this vaccine enhanced the production of IL4 (Th2-type) cytokine followed by generation of antibodies specific to the self-Aβ antigen [39]. Here for the first time we tested the safety and efficacy of mannan-Aβ_28 _conjugate in Tg2576 mouse model of AD. We demonstrated that, in the absence of any conventional adjuvant, mannan-Aβ_28 _conjugate enhanced the production of Th2-type anti-Aβ antibodies inhibiting Aβ plaque formation. However, the mannan-peptide conjugate induced higher levels of microhemorrhages compared to that in the brains of age-matched control animals.

## Methods

### Preparation of mannan-Aβ_28 _immunoconjugate

Mannan (*Saccharomyces cerevisiae*, Sigma, MO) was purified by passage over a Q Sepharose FF column (GE Healthcare) to remove residual nucleic acid. Mannan was activated [40] and conjugated to Aβ_28 _peptide, as previously described[39]. In brief, a 10 mg/ml solution of mannan was activated by addition of the organic cyanylating reagent 1-cyano-4-dimethylaminopyridinium tetrafluoroborate (CDAP) (25 μl/ml, 100 mg/ml in acetonitrile). After 30 sec, 25 μl/ml of aqueous 0.2 M triethylamine (TEA) was added. After another 2 min an equal volume of 0.5 M hexanediamine, pH 9, was added and the reaction allowed to proceed overnight and then dialyzed against water. The mannan concentration and the free amine content were determined as previously described [40]. The amino-mannan was bromoacetylated using NHS bromoacetate, and desalted by dialysis. Aβ_28 _peptide (5.0 mg/ml) with an N-terminal linker (n-CAGA) sequence synthesized by (Multiple Peptide Systems, San Diego, CA) was dissolved in 0.15 M HEPES, 2 mM EDTA, pH 7.3, and the free thiol content determined using Ellman's reagent. The peptide was combined with the bromoacetylated mannan at a molar ratio of 30 thiols/100 kDa of carbohydrate under a stream of nitrogen. After an overnight reaction, the solution was quenched by the addition of mercaptoethanol concentrated with an Ultra 4 (10 kDa cut-off) device (Millipore, MA), and free peptide removed by gel filtration on a Sepharose 12 column (GE Healthcare, NJ), equilibrated with 0.1 M HEPES, pH 8. The void volume peak was pooled. The peptide content was determined from the UV spectrum and its calculated extinction coefficient. The final product contained 50 μM peptide at a ratio of 5 moles of peptide per 100 kDa of mannan formulated in PBS.

To generate a control mannan-antigen conjugate, mannan was coupled with BSA via a thiol-ether linkage as described for Aβ_28 _peptide except that the reaction was quenched with ethanolamine before gel filtration on a S300HR column (GE Healthcare, NJ). The peak tube from the void volume was assayed by the Bradford method using BioRad protein reagent, with BSA as the standard. Finally, biotinylated mannan was prepared by reaction of biotin-NHS with the amino-mannan, and un-reacted biotin was removed by gel filtration.

### Immunization of mice and detection of anti-Aβ and anti-mannan antibodies

Based on our results with wild-type mice [39], we immunized young 4–4.5 mo-old Tg2576 (APPsw) mice [Tg (HuAPP695.K670N/M671L) 2576] [41,42] without pre-existing AD-like pathology [43], (n = 8, 3 males, 5 females), six times biweekly with 10 μg/mouse of mannan-Aβ_28 _conjugate, subcutaneously (s.c.). Control mice of the same age were either injected 6 times s.c. with mannan-BSA conjugate (n = 6, 3 males, 3 females), or remained non-immunized (n = 5, 2 males, 3 females). We collected sera on the 9^th ^day after the last injection, or at the end of the resting period (~11 months), and examined the humoral immune responses by ELISA. Anti-Aβ antibodies and their isotypes were detected in individual sera as previously described [39,44-46]. Of note, concentrations of anti-Aβ antibodies were calculated using a standard curve generated with 6E10 monoclonal antibody (Signet, MA). Also, using a similar ELISA technique we detected the titers of anti-mannan antibodies, although in these experiments streptavidin pre-coated plate wells (Express Biotech International, MD) were coated with 2 μg/ml biotinylated mannan as recommended by manufacturer before addition of immune or control sera.

### Dot blot assay

Binding of anti-Aβ_28 _antibody to different forms of Aβ_42 _peptide was demonstrated as described [47]. Briefly, 1 μl of monomeric, oligomeric or fibrillar forms of Aβ_42 _(45 μM each) were applied to a nitrocellulose membrane. After blocking and washing, the membranes were probed with sera from mice immunized with mannan-BSA, mannan-Aβ_28 _(1 μg/ml), anti-Aβ 20.1 monoclonal (1 μg/ml) antibody, or rabbit A11 polyclonal (1 μg/ml) antibody previously described by [48]. The membranes were incubated with appropriate HRP-conjugated anti-mouse (1:2000) or anti-rabbit (1:2000) antibody (Santa Cruz Biotechnology, CA). Blots were developed using Luminol reagent (Santa Cruz Biotechnology, CA) and exposed to Kodak X-Omat AR film.

### Immunohistochemistry

After the termination of the study, we obtained the brains of experimental and control mice (~18–18.5 months) and analyzed the impact of active immunization with mannan-Aβ_28 _on neuropathological changes as we previously described [47,49,50] Briefly, mice were halothane-anesthetized, perfused with PBS prior to removing the brains. Brains were fixed (4% paraformaldehyde, 48 hrs), and brain tissues were sectioned (40 μm thick) and stored at 4°C (PBS with 0.02% sodium azide). Staining of duplicates of coronal sections of experimental and control mouse brains, collected from 3 different planes of the brain about 1 mm apart, with monoclonal anti-Aβ_40 _or Aβ_42 _(Signet, MA) as we described [49].

The quantitative analysis of CAA was made as described by [51]. Briefly, CAA score (based on frequency and severity) was quantified on the sections immunostained with anti-Aβ_40 _throughout the neocortex. Six sections from each brain in the groups of mice immunized with mannan-Aβ_28_, mannan-BSA or non-immunized animals were analyzed. CAA frequency was calculated by counting the total number of Aβ-positive vessels in the set of sampled sections, while CAA severity was assessed using three severity grades: grade 1, Aβ immunoreactivity confined strictly to the vessel wall; grade 2, Aβ immunoreactivity in and around vessel wall with focal infiltration of the amyloid in the neuropil; and grade 3, extensive infiltration of amyloid into the neuropil with a complete amyloid coat around the vessel. The mean for all vessels was taken as CAA severity, and final CAA score was calculated by multiplying CAA frequency with CAA severity. All of the quantitations were done on both hemispheres by two independent investigators and yielded similar results.

Binding of the pooled immune sera from the group of mice immunized with mannan- Aβ_28 _to the 50 μm brain section of formalin-fixed cortical tissue of a 75-year-old woman with neuropathological and behavioural patterns typical to severe AD, was done as we described previously [45,47]. To demonstrate the specificity of binding of anti- Aβ_28 _antibodies to the brain tissue we blocked antisera from immune mice with the Aβ_1–15 _peptide at 5 μM concentration as described [47].

For detection of astrocytosis, we used cow glial fibrillary acidic protein, GFAP (DAKO, Denmark). To detect microgliosis, we used anti-mouse CD45 (LCA, Ly-5, Pharmingen, CA). Anti-C1q and anti-C3b antibodies (eBioscience, CA) were used to detect activation of complement as previously described [52]. Infiltration of T cells was analyzed using anti-CD3-ε (Santa Cruz Biotechnology, CA) antibody. The tissues from all animals within a given experimental group were processed in parallel. Hydrogen peroxide-quenched and blocked sections were incubated with primary antibody overnight at 4°C. Sections were then washed and incubated with appropriate biotinylated secondary antibodies against corresponding species. Colour developed via ABC and DAB (3,3'-diaminobenzidine) substrate kits (Vector, CA). Images of the immunostaining were captured using a Sony high-resolution CCD video camera (XC-77), and quantification of staining was performed using NIH image 1.59b5 software. For every animal, eighteen images (525 × 410 μm each) of the frontal parietal region in the cortex area of 2 adjacent sections were captured with 20× objective. Samples consisted of nine images from the superficial layer and the remaining nine from the deep layer. NIH imaging was used to analyze the area occupied by amyloid plaques, as well as CD45- and GFAP-immunoreactivity. The threshold for the detection of immunoreactivity was established and then held constant throughout the image analysis. Additionally, we performed a visual inspection of hippocampal and cortical regions of all stained sections in a blinded fashion (observer had no knowledge of the treatment group); a mean semi-quantitative score was independently determined for each slide by 2 observers.

#### Histochemistry

Determination of cored plaques was accessed using Thioflavin-S (ThS) according to a standard protocol from the IBAD, UCI. Briefly, sections from immunized and control animals were mounted on Vectabond-coated slides (Vector Labs, CA). Sections were then washed with Tris buffer (0.1 M Tris buffered saline at pH 7.5) and stained with 0.5% ThS (Sigma, MO) in 50% ethanol for 10 min. Finally, sections were washed in 50% ethanol, and twice in Tris buffer, then dried and coverslipped with Vectashield (Vector Labs, CA). ThS-positive plaques were counted by visual inspection of cortical and hippocampal regions of all stained sections in a blinded fashion; a mean semi-quantitative score was independently determined for each slide by 2 observers.

Determination of microhemorrhages was performed using Prussian blue staining of ferric acid according to a standard protocol from IBAD, UCI. Briefly, duplicates of coronal sections of experimental and control mouse brains, 40 μm thick, collected from 3 different planes of the brain (about 1 mm apart), were mounted on Vectabond coated slides (Vector Labs, CA) and stained with Prussian blue working solution, a mixture of freshly made 5% Potassium hexacyanoferrate (II) trihydrate, and 5% hydrochloric acid (all from Sigma, MO). After 30 min all sections were rinsed in water, counterstained with Nuclear Fast Red (Sigma, MO), dehydrated and covered using DPX (BDH Laboratory Supplies, England). The hemorrhage profiles (hemosiderin stain) were counted by two independent observers, and the average number of Prussian blue-positive deposits was calculated for each brain section.

For detection of co-localization of Aβ accumulation and complement markers in vasculature we used combination of two methods described above. First, sections were glass mounted, peroxidase-quenched and stained with anti C1q and C3b antibodies (eBioscience, CA) followed by biotinylated secondary antibodies and streptavidin-Alexa Fluor 555 (Thermo Fisher Scientific, IL). After that, ThS staining was performed as described above. Confocal images were collected on an Olympus IX70 inverted microscope and a BioRad Radiance 2000 Laser Scanning System using 70× objective for image analysis.

### Statistical Analysis

To assess the differences in antibody titers, plaque areas, glial immunoreactivity, frequency of microhemorrhages and CAA in experimental mice versus the control groups, a two-tailed Students's t-tests were performed using Graph Pad Prism 3.03 software.

## Results

### Mannan-Aβ_28 _immunoconjugate induced potent anti-Aβ antibody responses in the Tg2576 mouse model of AD

**Figure 1:**
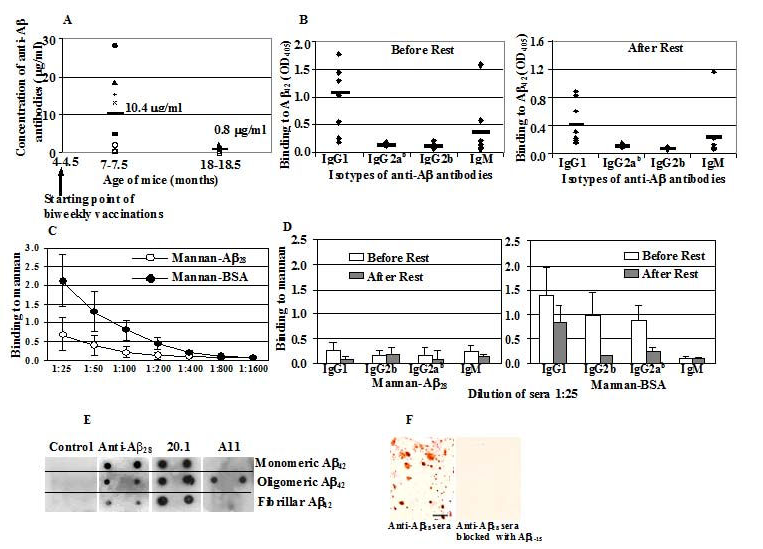
**Mannan-Aβ_28 _immunoconjugate induces potent levels of anti-Aβ and low levels of anti-mannan antibodies in Tg2576 mice**. A. Vaccinated mice 7–7.5 months of age produced on average 10.4 μg/ml of anti-Aβ antibodies (approximate titers ≥ 1:10,000) after 6 immunizations. At termination, when mice reached 18–18.5 months of age they still produced low levels of anti-Aβ antibodies (0.8 μg/ml or approximate titers ≥ 1:800). Noteworthy, mice immunized with mannan-BSA did not produce detectable levels of anti-Aβ antibodies (data not shown). B. Mannan-Aβ_28 _induced primarily IgG1 isotype antibodies, indicative of a Th2 type humoral immune response before resting period (sera were collected on 9^th ^day after the last boost) and after resting period (sera were collected after 11 months). One vaccinated mouse also induced high level of IgM anti-Aβ antibodies. C. After 6 injections vaccinated mice produced low titers of anti-mannan antibodies. Of note, the titers of anti-mannan antibodies dropped dramatically during the intervening 11 months following the final injection (data not shown). (D). Mannan-Aβ_28 _as well as mannan-BSA induced only low levels of anti-mannan antibodies predominantly of IgG and IgM isotypes in mice immunized with mannan-Aβ_28_, and IgG isotypes for mice immunized with mannan-BSA. E. Anti-Aβ_28 _antibodies bind to all species of Aβ_42 _peptide. One μl of Aβ_42 _fibrils, oligomers and monomers (45 μM each) were applied in duplicate to a nitrocellulose membrane and probed with sera from mannan-BSA immunized mice (control), sera from mice immunized with mannan-Aβ_28_, anti-Aβ 20.1 monoclonal antibody and rabbit A11 antibody shown to bind oligomeric forms of Aβ_42_. (F). Binding of immune mannan-Aβ_28 _sera to the AD brain section. Binding of antisera was blocked by Aβ_1–15 _peptide. Original magnification, 20×, scale bar = 100 μm.

Previously, we have demonstrated that 10 μg of human mannan-Aβ_28 _peptide conjugate enhanced Th2 type anti-Aβ antibody production in wild-type mice[39]. In the current study we first analyzed the immunogenicity of mannan-Aβ_28 _immunoconjugate in the Tg2576 mouse model of AD in which Aβ_28 _is a self-antigen. After six biweekly immunizations with the same low dose (10 μg) of mannan-Aβ_28 _antigen, 5 out of 8 Tg2576 mice generated robust anti-Aβ antibody production, whereas the remaining mice expressed less than 5 μg/ml of antibodies (Figure 1A). Average anti-Aβ antibody concentrations were 10.4 μg/ml, equal to titers of ≥ 1:10,000. The humoral immune response generated by the mannan-Aβ_28 _immunogen was long lasting since the anti-Aβ antibody concentration remained readily detectable (0.8 μg/ml or approximately a titer of ≥ 1:800) in vaccinated animals that were held free of immunizations for 11 months (Figure 1A). Antibody isotyping can be used as an indirect measure of the contribution of Th1 (IgG2a) and Th2 (IgG1) cytokines to the humoral response[53]. Additionally, the subclass of anti-Aβ antibodies may correlate with their therapeutic potential[11,54]. Thus, we measured the production of IgG1, IgG2a^b^, IgG2b, and IgM anti-Aβ antibodies in the sera of vaccinated Tg2576 mice, demonstrating that mannan-Aβ_28 _induced predominantly IgG1, although two mice also produced significant amount of IgM antibody (Figure 1B). Interestingly, the isotypic profile of anti-Aβ antibodies was similar at 9 days and 11 months after the last boost (Figure 1B), and the ratios of IgG1 to IgG2a^b ^antibody in these mice were 8.2 and 5.25, respectively. Thus, immunization with the Aβ_28 _immunoconjugate induced long-lasting therapeutically potent anti-Aβ antibodies in Tg2576 mice, and the humoral immune response was Th2-polarized, similar to the observations after the conjugation of mannan with other peptide immunogens [33,37,38] or immunizations of wildtype mice with mannan-Aβ_28_[39].

Mannan was previously shown to be a weak T-independent antigen, however because the mannan was conjugated to a peptide (Aβ) or a protein (BSA), both of which contain T helper cell epitopes that can be used to promote the production of anti-mannan antibodies, we analyzed the antibody response to mannan induced by immunizations with mannan-Aβ_28 _or mannan-BSA conjugates. As we expected, both groups of mice induced only low titers of anti-mannan antibodies at day 9 after the last boost (Figure 1C). The anti-mannan antibodies were of the IgG and IgM isotypes in mice immunized with mannan-Aβ_28 _and predominantly of IgG isotypes in mice immunized with mannan-BSA (Figure 1D). It was not surprising that both immunoconjugates induced IgG anti-mannan antibodies because Aβ_28 _[44] and BSA possess T helper epitope/s that make mannan-peptide/protein conjugate a T-dependent antigen.

In the light of recent data that oligomeric forms of Aβ_42 _peptide are the most toxic forms, we analyzed the binding ability of immune and control sera to Aβ monomers, oligomers and fibrils by a dot blot assay. Sera from mannan-Aβ_28 _immunized, but not from mannan-BSA immunized mice, bound to all forms of Aβ_42_, although binding was better to the monomeric and oligomeric forms than to fibrillar Aβ_42 _(Figure 1E). We also demonstrated that immune sera bound to Aβ plaques in the brain tissue from an AD clinical case. This binding was specific since pre-incubation of sera with the Aβ_1–15 _peptide resulted in its complete inhibition (Figure 1F).

### Immunization with mannan-Aβ_28 _inhibits AD-like amyloid pathology in the brain

**Figure 2:**
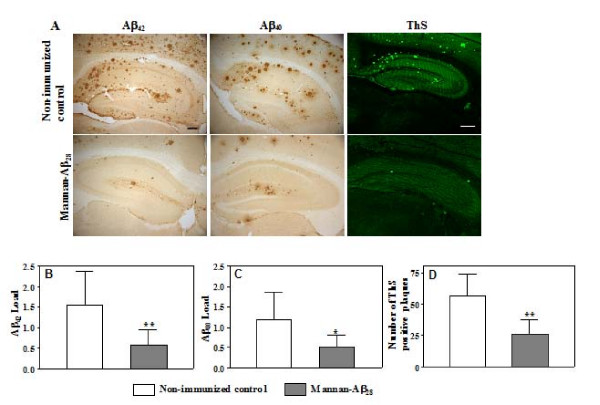
**Vaccination with mannan-Aβ_28 _attenuates amyloid deposition in the cortical and hippocampal regions of brains**. A. Representative images of hippocampal region from non-immunized controls and mice immunized with mannan-Aβ_28 _showing reduction of Aβ_42 _and Aβ_40 _amyloid burden or cored amyloid plaques. Aβ deposits were stained with anti-Aβ_42_, anti-Aβ_40 _antibodies, and with ThS. Original magnifications, 5× for anti-Aβ_42/40 _staining and 4× for ThS (scale bars = 200 μm). B, C, D. Image analysis of Aβ_42 _(B), Aβ_40 _(C) staining, and number of ThS-positive cored plaques (D) in cortex of 18–18.5 mo old Tg2576 non-immunized mice and mice immunized with mannan-Aβ_28_. Vaccinated mice showed significant reduction in Aβ_42 _(P = 0.0075), Aβ_40 _(P = 0.0103) burden, and in number of ThS-positive cored plaques (P = 0.0019) compared to that in non-immunized control group. Bars represent mean ± SE from n = 5 in the group of non-immunized mice, and n = 8 in the group of mice immunized with mannan-Aβ_28_.

Next, we analyzed the extent of AD-like pathology in brains of vaccinated Tg2576 mice (18–18.5 months old). As controls, we used Tg2576 mice of the same age injected with mannan-BSA, or non-immunized group of animals. Image analysis of anti-Aβ_40 _and anti-Aβ_42 _antibody-stained parietal cortical sections demonstrated a significant reduction of amyloid plaques in brains of mice vaccinated with mannan-Aβ_28 _compared with similar brain staining of non-immunized mice (Figure 2) or animals injected with mannan-BSA (data not shown). While staining with anti-Aβ_40 _and anti-Aβ_42 _antibodies detected both cored and diffuse plaques, ThS binds only to cored amyloid deposits. Therefore, we compared image analyses with ThS staining of parietal cortical brain sections from vaccinated and control Tg2576 mice. We found that mice actively immunized 11 months earlier with the mannan-Aβ_28 _conjugate had significantly reduced depositions of cored amyloid plaques (Figure 2D). A similar reduction in Aβ_40 _and Aβ_42 _amyloid burden, as well as cored plaques was observed in the hippocampus of vaccinated mice compared with control non-immunized animals (Figure 2A). Of note, we observed no changes in Aβ depositions by immunohistochemistry or histochemistry in the brains of mice immunized 6 times with mannan-BSA compared to non-immunized control (data not shown). Collectively, these results demonstrate that the mannan-Aβ_28 _immunogen induced therapeutically potent anti-Aβ antibodies, predominantly of the IgG1 isotype, that effectively reduced the deposition AD-like plaque pathology in 18–18.5 months old Tg2576 mice.

### Immunizations with mannan-Aβ_28 _reduced glial activation in brains of Tg2576 mice

**Figure 3:**
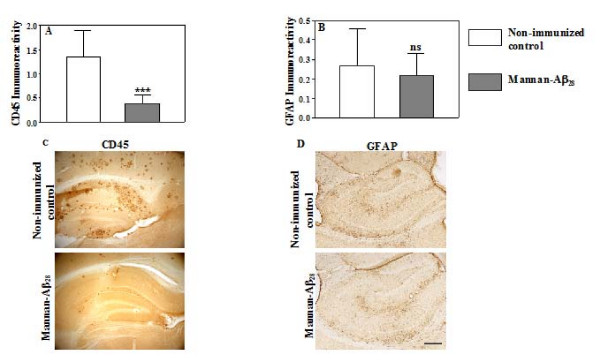
**Effects of vaccination with mannan-Aβ_28 _on microglial and astrocyte activation in Tg2576 mice**. Image analysis of cortical sections performed after staining with anti-CD45 (A) and anti-GFAP (B) antibodies of brain sections from non-immunized controls and mice immunized with mannan-Aβ_28_. Significantly less microglial activation (P = 0.0003) and lower level of astrocytosis (P = 0.3263) were detected after immunization with mannan-Aβ_28 _compared to non-immunized control mice. Representative pictures of staining with anti-CD45 (C) and anti-GFAP (D) antibodies showing a decrease in immunoreactivity in hippocampal areas of brain sections of mice immunized with mannan-Aβ_28 _versus control non-immunized mice. Original magnifications: 5×, scale bar = 200 μm.

It was previously reported that following active and passive Aβ-immunotherapy, microglia activation in brains of aged APP/Tg mice was attenuated [55-59]. In the current study, we investigated the relationship between a reduction in fibrillar amyloid deposition and the state of microglial activation in the brains of Tg2576 mice. After eleven months mannan-Aβ_28 _vaccination there was significantly less microglia activation as detected both in the frontal cortex and in the hippocampus using anti-CD45 antibody immunostaining and image analysis (Figure 3A, C). In fact, in the hippocampus areas of vaccinated mice we detected only a few cells weakly stained for CD45, while similar brain areas of the age-matched non-immunized Tg2576 mice (Figure 3C) or mannan-BSA (data not shown) were stained intensively for CD45.

It was previously shown that Aβ immunotherapy may reduce neuritic dystrophy and astrogliosis, along with reversing memory deficits in mouse models of AD [11,60,61]. To determine the role of vaccination with mannan-Aβ_28 _in the reduction of astrocytosis, we stained brains of vaccinated and non-immunized control mice with anti-GFAP antibody. Image analyses of parietal cortical tissues demonstrated that brains of vaccinated mice were stained less robustly than those of control animals. Although, there was a trend toward less stimulation of astrogliosis, it did not reach significance in the cortex (Figure 3B), a similar trend was also observed in the hippocampus region of brains from vaccinated mice (Figure 3D).

### Vaccination with mannan-Aβ_28 _immunoconjugate induced microhemorrhage

**Figure 4:**
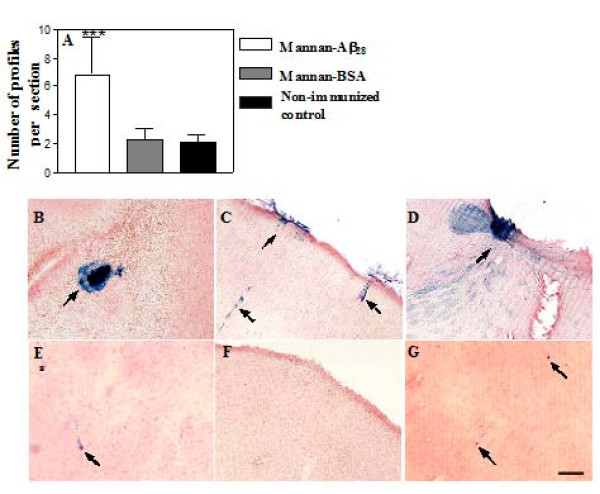
**The incidence and severity of vascular microhemorrhages increased following mannan-Aβ_28 _immunizations**. A. Quantification of Prussian blue staining based on the total number of positive profiles per section for mice injected with mannan-Aβ_28_, mannan-BSA, or non-immunized group. The levels of microhemorrhages were increased in vaccinated Tg2576 mice (n = 8) to 6.81 ± 2.6 compared to that of 2.31 ± 0.84 in mice immunized with mannan-BSA (n = 6), and 2.07 ± 0.58 in non-immunized animals (n = 5). *** Indicates *P *< 0.001. B-G. Examples of microhemorrhage severity in the brain sections of mice injected with mannan-Aβ_28 _(B, C, D), mannan-BSA (E, F), or non-immunized animals (G). B. A focal microhemorrhage (arrow) at the cortical-subcortical junction, below the gray-white matter border, lateral to hippocampus. C. Representative images of the Prussian blue-positive blood vessels (arrows) most prominent in the meningeal, superficial parenchymal and cortical vessels. D. A focal hemorrhage in the area of anterior commissure. E. A single microhemorrhage (arrow) in the white matter region. F. Microhemorrhage-free cortical and leptomeningeal areas. G. Two microhemorrhages (arrows) in the cortical and white matter regions. Original magnification, 10×, scale bar = 100 μm.

**Figure 5:**
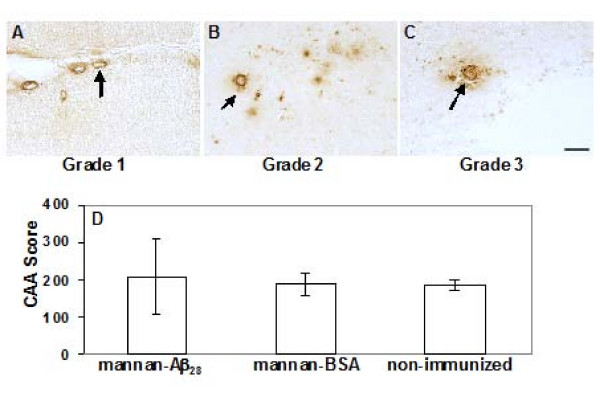
**Semi-quantitative analysis of CAA in the neocortex areas of animals immunized with mannan-Aβ_28_, mannan-BSA, or non-immunized mice**. A, B, C. Examples of CAA severity grades: grade 1, Aβ immunoreactivity confined strictly to the vessel wall (A); grade 2, Aβ immunoreactivity in and around vessel wall with focal infiltration of the amyloid in the neuropil (B); grade 3, extensive infiltration of amyloid into the neuropil with a complete amyloid coat around the vessel (C). Original magnification 20×, scale bar = 50 μm. D. The CAA score detected in the neocortex of mice vaccinated with mannan-Aβ_28_, was similar to that detected in neocortex of animals injected with mannan-BSA or non-immunized mice (P > 0.05). Bars represent mean ± SE from n = 5 in the group of non-immunized mice, n = 6 in the group of mannan-BSA immunized group, and n = 8 in the group of mice immunized with mannan-Aβ_28_.

Previous studies have shown that passive immunotherapy with high doses of anti-Aβ antibodies may induce microhemorrhage in aged APP/Tg mice [18,21]. More recently, it was reported that multiple (10 times) immunizations of APP+PS1 mice with high doses of fibrillar Aβ_42 _formulated in conventional adjuvant (FCA/FIA) could also increase microhemorrhage in 20 months old mice [22]. Interestingly, in PDAPP mice passive vaccination with anti-Aβ antibodies cleared vascular amyloid, but high doses of antibodies induced microhemorrhages that are limited to focal perivascular sites [62]. Here, we analyzed microhemorrhage in 18–18.5 months-old Tg2576 mice, vaccinated with low doses of mannan-Aβ_28_, for 11 months prior to sacrifice, by hemosiderin staining with Prussian blue. Numbers of positive profiles per section of the brains of mannan-Aβ_28 _vaccinated animals were compared with that in the brains of control age-matched animals immunized with mannan-BSA, or non-immunized mice. We observed low numbers of microhemorrhage in both non-immunized and mannan-BSA injected control aged mice. In contrast, following vaccination with mannan-Aβ_28_, we detected significantly (*P *< 0.001) higher numbers of microhemorrhage (Figure 4A). Additionally, we observed larger microhemorrhage in mice vaccinated with mannan-Aβ_28 _(Figure 4B, C, D), than in mannan-BSA (Figure 4E, F), or non-immunized control animals (Figure 4G). Previously, it was demonstrated that passive Aβ-immunizations resulted in reduction of amyloid deposition, but also increased incidence of microhemorrhage associated with increased CAA in humans [14] and APP/Tg mice [20]. Thus, we undertook a semi-quantitative analysis of the CAA in the cortical brain sections of mice injected with mannan-Aβ_28_, mannan-BSA, or non-immunized animals. CAA score was calculated based on the frequency and severity of the Aβ immunoreactivity confined strictly to the vessel wall, around vessel wall with focal infiltration of the amyloid in the neuropil, or extensively infiltrated into the neuropil with a complete amyloid coat around the vessel as it is shown in Figure 5A, B, C. Using this approach we calculated CAA in neocortical areas of experimental and control animals and demonstrated that the CAA score was almost equal in mice vaccinated with mannan-Aβ_28_, mannan-BSA or non-immunized control (P > 0.05, Figure 5D). Of note, we did not quantitate CAA in the meninges in this study, because the above mentioned standard Aβ burden measurements made in the parenchyma and normalized to brain region were not applicable to the meningeal location of vascular Aβ deposits.

**Figure 6:**
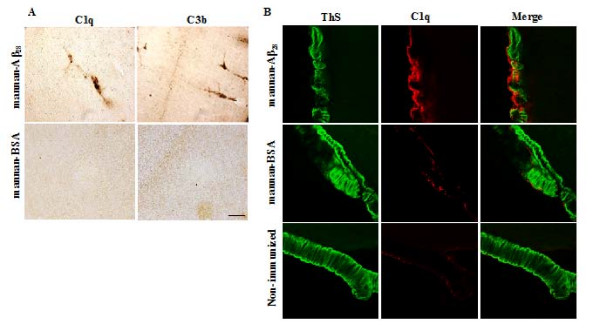
**Immunostaining of neocortex for C1q and C3b components of complement, and concomitant staining of meninges for C1q and ThS**. A. Representative images of cortex areas of coronal brain sections of mice immunized with mannan-Aβ_28 _or mannan-BSA, immunostained with antibodies specific for C1q and C3b components of complement. Original magnification, 20×, scale bar = 50 μm. B. Representative images of colocalization of vascular fibrillar Aβ detected with ThS (green) with C1q component of complement (red) in the meninges of coronal brain sections of mice immunized with mannan-Aβ28, mannan-BSA or non-immunized animals. Original magnification, 70×.

Since the activation of the complement cascade by antibody-Aβ immune complexes at sites of cerebral amyloid angiopathy (CAA) may be one possible cause of microhemorrhage, it was of interest to analyze the opsonization of sites of microhemorrhage with C1q and C3 proteolytic fragments, C3b, complement components in mice immunized with mannan-Aβ_28_. We detected the accumulation of these complement proteins in the cerebral vasculature of immunized, but not control mice immunized with mannan-BSA (Figure 6A). In the meningeal vessels, in the group of mannan-Aβ_28 _immunized animals we have demonstrated partially cleared ThS-stained vascular fibrillar Aβ deposits abundantly decorated with C1q component of complement, while only trace amount of C1q was co-localized with compact Aβ load in the meninges of mannan-BSA immunized group, and practically none of the C1q was detected in the Aβ-laden meninges of non-immunized animals (Figure 6B). The similar co-localization pattern was shown for C3b component of complement (data not shown).

## Discussion

Anti-Aβ immunotherapy is one strategy for the inhibition/reduction of soluble and insoluble amyloid-β deposits that likely play a causal role in the onset and progression of AD [5,6]. Several pre-clinical studies with mouse models of AD have demonstrated the ability of Aβ vaccination to prevent amyloid deposition in the brain. Thus, active immunization with Aβ [7,63], as well as passive immunization intraperitoneally [11], or direct to the brain application of anti-Aβ antibodies[64], resulted in reduction of the amyloid burden in the brain. Importantly, active immunizations prevented cognitive decline [8,9], and passive immunizations were shown to reverse the memory loss in aged Tg2576 mice [65]. While these and other studies did not report any adverse events in AD mouse models immunized with fibrillar Aβ_42_, data from the AN-1792 vaccine trial suggest that the adverse reactions to Aβ_42_-immunotherapy were not due to the humoral antibody response, but rather to the cell-mediated autoimmune response, possibly exacerbated by the reformulation of adjuvant, QS21 supplemented with polysorbate-80, which induced a Th1 type immune response in patients that received the AN1792 vaccine [13-16,63,66-69]. Thus, the goal of this pre-clinical study was to employ a molecular adjuvant, mannan, that induces a Th2-type humoral immune response specific to Aβ peptide, and test the efficacy of vaccination on AD-like pathology in aged Tg2576 mice.

Previously, mannan was shown to enhance both humoral and cellular immunity against various immunogens conjugated to mannan. Multiple mechanisms likely contribute to the adjuvant properties of mannan conjugates, including activation of complement, enhancing uptake and presentation of the conjugated antigen, as well as by providing a stronger signal to antigen-specific B cells by simultaneous triggering of B-cell receptors and CD21/CD35 molecules [26-28,31-35,70-75]. Notably, under certain conditions mannan was shown to induce predominantly Th2 polarized anti-inflammatory immune responses to a conjugated non-self peptides [33,34,37,38], a response which would presumably be beneficial to an anti-Aβ vaccine. We previously reported that Aβ_42 _possesses B and T cell antigenic epitopes, both of which are contained within the Aβ_28 _peptide in mice [44]. In addition, our previous studies in wild-type mice using mannan conjugated to Aβ_28 _peptide was able to induce potent and highly polarized Th2 phenotype anti-Aβ antibody responses [39]. In the current study, we immunized anti-Aβ hyporesponsive Tg2576 mice with mannan-Aβ_28 _conjugate prior to the onset of amyloid plaque pathology in the brains of Tg2576 mice [43,76]. Mice were immunized six times with low doses (10 μg per mouse) of mannan-Aβ_28 _conjugate and the titer of anti-Aβ antibodies were assessed by ELISA. The antibodies induced by the mannan-Aβ_28 _conjugate were predominantly of the IgG1 isotype and elevated titers were maintained for 11 months without further immunizations (Figure 1A, B). Thus, we demonstrated that our mannan-Aβ_28 _immunoconjugate induced long-lasting and Th2-polarized anti-Aβ humoral immune responses in Tg2576 mice in the absence of a conventional adjuvant, such as alum or CFA. The humoral immune response induced by mannan-Aβ_28 _was similar to that reported after multiple injections of 5 to 10 times higher doses of fibrillar Aβ_42 _formulated in strong Th1 or Th2 conventional adjuvants [7,8,44,77-79].

We examined the effect of mannan-Aβ_28 _vaccination on the neuropathology in 18–18.5 months old mice, and showed a significant reduction in both the Aβ_40 _and Aβ_42 _burden, as well as in the number of ThS positive cored plaques in the parietal cortical and hippocampal regions (Figure 2) of the brains from mice immunized 11 months earlier. Of note, previously we demonstrated that Aβ load was significantly reduced in the brains of Tg2576 mice with a high concentration of serum anti-Aβ_1–11 _antibody (>50 μg/ml), whereas moderate concentration (5–50 μg/ml) of this antibody resulted in a trend toward a reduction in Aβ deposits, however it did not reach significance [49]. In the current study antibody specific to Aβ_28 _even at a moderate concentration (Figure 1A) significantly reduced both Aβ_40 _and Aβ_42 _deposits. This difference in potency of anti-Aβ antibody may be due not only to the different antigen configurations, but also to the starting point of immunization. More specifically, immunization with mannan-Aβ_28 _was started in mice without pre-existing pathology (preventive vaccination strategy), whereas in previous study [49] immunization was initiated in mice with established AD-like pathology (therapeutic vaccination strategy). Of note, we did not detect any CD3-positive T cells in the brains of mice immunized with mannan-Aβ_28 _(data not shown). Thus, inhibiting Aβ deposition in immunized Tg2576 mice was not associated with any adverse events, except for the increase in microhemorrhages.

The first two case reports from the AN-1792 clinical trial both reported meningoencephalitis with lymphocyte infiltration in the brain [15,16]. Additionally, the third case report demonstrated increased CAA without clinical presentation of meningoencephalitis, however there was noticeable perivascular T cell infiltration [14]. It is worth mentioning, that a high degree of CAA typically indicates that hemorrhages are present in the brain [80,81]. However, only the case report from an AD patient from the Barcelona cohort of the AN-1792 trial found fully developed hemorrhages [16]. The data on anti-Aβ immunotherapy in experimental mouse models of AD are somewhat different. Normally, rodents do not develop Aβ-CAA, except several APP transgenic mice models, including aged Tg2576 mice developing vascular amyloid in addition to the significant parenchymal amyloid [82,83]. Importantly, several studies with aged APP/Tg mice reported cerebral vascular microhemorrhages after passive transfer of high doses of anti-Aβ antibodies [18,20,84]. Additionally, it was shown that 3D6 antibody specific to N-terminal region of amyloid, but not antibody against Aβ_16–23 _(266) prevented or cleared vascular Aβ in a dose-dependent manner, but high concentration of this antibody was associated with microhemorrhages in focal perivascular sites [62]. Recently, it was reported that active immunizations with a high dose of fibrillar Aβ_42 _plus CFA/IFA also increased microhemorrhage in aged APP/Tg mice [22]. However, only one study reported meningoencephalitis in an APP/Tg mouse model passively immunized with repeated injections of high doses of monoclonal anti-Aβ antibodies (1 mg within 16 days). Although only 1 out of 21 experimental mice were affected, the authors nonetheless suggested that an inflammatory mechanism might be triggered by antibody binding to cerebral CAA, which in turn may enhance activation of autoreactive T cells, or lead to local disruption of the BBB [85]. Accordingly, in the current study we sought to investigate whether immunization with a mannan-Aβ_28 _conjugate might induce infiltration of T cells or increase the incidence of microhemorrhages, and CAA. While an examination of the brains did not reveal meningoencephalitis induced by the presence of T lymphocytes (data not shown), we observed an increased incidence and the severity of microhemorrhages in Tg2576 mice vaccinated with mannan-Aβ_28 _(Figure 4). CAA-associated microhemorrhage in aged APP/Tg mice, was ameliorated with deglycosylation of antibodies used for passive immunization [21], implicating the potential harmful effect of mannan as the trigger of adverse vascular events. Thus, we attempted to demonstrate CAA-associated microhemorrhages in vaccinated animals with anti-Aβ antibodies. We have started with examination the meningeal and cortical vascular Aβ burden, because similar to human vascular Aβ, in mice it is first detectable within leptomeninges, followed mostly by cortical, hippocampal and thalamic areas [86,87]. By 12 months of age, Aβ deposits in Tg2576 mice are present in the walls of leptomeningeal and cortical blood vessels, and at 15 months, the percentage load of Aβ-CAA in these areas is about 1.46% [88]. As mice in our study approached 18 mo of age, the CAA score in neocortex appeared to be similar in all groups of experimental and control mice (Figure 5D). Of note, in this study we had not quantified the difference in the leptomeninges of experimental animals precisely, due to non-applicability of the standard percentage area measurements of Aβ made in parenchyma and normalized to brain region to the measurements of the meningeal location of vascular Aβ deposits.

Although the perivascular macrophages express high levels of mannose receptors [29], it is unlikely that mannan fused with Aβ_28 _could cross the BBB and activate these cells. Of note, infiltration of perivascular macrophages has been detected predominantly in gray matter, with the corpus callosum having very few cells, although substantial microhemorrhages in our study were detected among other areas, both in gray matter and in corpus callosum. In addition, immunization with mannan-BSA did not elevate the level of hemorrhages compared with low profiles observed in non-immunized mice (Figure 4).

Notably, titers of the anti-mannan antibodies in mannan-BSA group were higher than that in mice vaccinated with mannan-Aβ_28 _(Figure 1C). Although these data indicate that anti-mannan antibodies did not contribute directly to the adverse cerebral vascular events, it is possible that they may contribute to an increase in BBB permeability, thereby increasing access of anti-Aβ antibody to perivascular and parenchymal spaces in mice vaccinated with mannan-Aβ_28_. Importantly, we previously demonstrated that mannan-Aβ_28 _or Aβ_28 _alone did not induce microhemorrhage in the brains of wild-type mice [39].

Thus, we hypothesize that a potential cause for the incidences of microhemorrhage detected in Tg2576 mice immunized with mannan-Aβ_28 _may be anti-Aβ antibody binding to Aβ in cerebral vasculature. If so, one potential culprit in the microhemorrhage may be the activation of the complement cascade by antibody-Aβ immune complexes at sites of CAA [89,90]. Our data demonstrating that C1q and C3b are found in the cortical brain sections of mice immunized with mannan-Aβ_28_, but not control animals (Figure 6A) indicate that binding of antibody-Aβ to Aβ deposits in the cerebral vasculature may in fact induce microhemorrhages. Of note, we demonstrated that the level of decoration of ThS-stained vascular fibrillar Aβ with complement components C1q (Figure 6B) and C3b (data not shown) was highest in the meninges of mice immunized with mannan-Aβ_28_. In this group, β-amyloid deposits had altered morphology suggestive of ongoing clearance, and were heavily decorated with C1q component of complement. On the contrary, very small or practically absent C1q profiles were co-localized with substantial and unaltered Aβ load of the meninges of BSA-mannan-immunized or non-immunized groups, respectively (Figure 6B). Importantly, recently it was shown that microhemorrhages in PDAPP mice passively immunized with the 3D6 antibody, being dose-responsive, co-localized with the vascular amyloid accumulations having altered Aβ morphology [62].

It is noteworthy that the microhemorrhages triggered by anti-Aβ antibodies may play an important role in reduction of AD-like pathology, which we observed in aged Tg2576 mice vaccinated with mannan-Aβ_28_. This seems plausible, since only low concentrations of free anti-Aβ antibodies were detected in the sera of vaccinated mice after 11 months without further boosting with the mannan-Aβ_28_, and mannan-BSA did not induce microhemorrhages. Although we observed less total glial activation in the brains of mice vaccinated with mannan-Aβ_28_than in the brains of control animals (Figure 3), it is possible that areas affected by microhemorrhages may have higher CD45 and GFAP immunoreactivity. Future studies of the underlying mechanisms responsible for the antibody-induced microhemorrhages in mice may be useful for avoiding these potentially adverse events in AD patients receiving anti-Aβ immunotherapy.

## Conclusion

Our novel vaccine candidate based on Aβ_28 _conjugated to mannan, as the molecular adjuvant, induced production of anti-Aβ antibodies in Tg2576 mice, which have been shown to be hyporesponsive to immunization with Aβ self antigen, in the absence of conventional adjuvant. As expected, the mannan-Aβ_28 _conjugate induced the generation of Th2-type anti-Aβ antibodies, which significantly attenuated amyloid deposition in old Tg2576 mice. However, there were increased levels of cerebral microhemorrhage in mannan-Aβ_28 _immunized mice, which did not appear to be due to the anti-mannan antibody response induced by the immunoconjugate, because mice immunized with mannan-BSA generated higher titers of anti-mannan antibodies than mice immunized with mannan-Aβ_28 _and they did not show elevated levels of microhemorrhage. Whether the anti-mannan antibodies increased the permeability of the BBB thus allowing elevated levels of anti-Aβ antibodies entry into cerebral perivascular and parenchymal spaces contributed to the increased incidence of microhemorrhages remains to be investigated in future studies.

## Competing interests

The authors declare that they have no competing interests.

## Authors' contributions

IP helped perform the immunizations of mice, brain collection and preparation of sections, immunohistochemical and histochemical analysis of brain sections and drafted the manuscript. AG participated in the design of the study, carried out blood collection, performed dot blot analysis for characterization of anti-Aβ antibody and helped to draft the manuscript. MM participated in the design of study and performed the statistical analysis. GM performed the quantification of staining using NIH image 1.59b5 software, conducted the ThS staining of brain sections. NM carried out ELISA for determination of anti-Aβ and anti-mannan antibody. RA helped to immunize mice and to prepare brain sections. VV analysed the activation of C1q and C3b in the brains of mice. AK helped to analyze antibody responses and to prepare figures. AL carried out the generation of mannan-Aβ_28 _and mannan-BSA conjugates and helped in the penultimate version of the manuscript. MGA conceived the study, helped in the design of the experiments and assisted in the preparation of the text and the figures in the manuscript. DHC conceived the study, designed and coordinated the experiments and helped in the drafting and editing of the manuscript. He was responsible for the final version of the manuscript. All authors read and approved the final manuscript.

## References

[B1] Price DL, Sisodia SS (1994). Cellular and molecular biology of Alzheimer's disease and animal models. Annu Rev Med.

[B2] Selkoe DJ (1991). Amyloid protein and Alzheimer's disease. Sci Am.

[B3] Selkoe DJ (1994). Alzheimer's disease: a central role for amyloid. J Neuropath and Exp Neurology.

[B4] Esler WP, Wolfe MS (2001). A Portrait of Alzheimer Secretases-New Features and Familiar Faces. Science.

[B5] Hardy JA, Higgins GA (1992). Alzheimer's disease: the amyloid cascade hypothesis. Science.

[B6] Hardy J, Selkoe DJ (2002). The amyloid hypothesis of Alzheimer's disease: progress and problems on the road to therapeutics. Science.

[B7] Schenk D, Barbour R, Dunn W, Gordon G, Grajeda H, Guido T, Hu K, Huang J, Johnson-Wood K, Khan K, Kholodenko D, Lee M, Liao Z, Lieberburg I, Motter R, Mutter L, Soriano F, Shopp G, Vasquez N, Vandevert C, Walker S, Wogulis M, Yednock T, Games D, Seubert P (1999). Immunization with amyloid-beta attenuates Alzheimer-disease-like pathology in the PDAPP mouse [see comments]. Nature.

[B8] Morgan D, Diamond DM, Gottschall PE, Ugen KE, Dickey C, Hardy J, Duff K, Jantzen P, DiCarlo G, Wilcock D, Connor K, Hatcher J, Hope C, Gordon M, Arendash GW (2000). A beta peptide vaccination prevents memory loss in an animal model of Alzheimer's disease. Nature.

[B9] Janus C, Pearson J, McLaurin J, Mathews PM, Jiang Y, Schmidt SD, Chishti MA, Horne P, Heslin D, French J, Mount HT, Nixon RA, Mercken M, Bergeron C, Fraser PE, St George-Hyslop P, Westaway D (2000). A beta peptide immunization reduces behavioural impairment and plaques in a model of Alzheimer's disease. Nature.

[B10] Chen G, Chen KS, Knox J, Inglis J, Bernard A, Martin SJ, Justice A, McConlogue L, Games D, Freedman SB, Morris RGM (2000). A learning deficit related to age and beta-amyloid plaques in a mouse model of Alzheimer's disease. Nature.

[B11] Bard F, Cannon C, Barbour R, Burke RL, Games D, Grajeda H, Guido T, Hu K, Huang J, Johnson-Wood K, Khan K, Kholodenko D, Lee M, Lieberburg I, Motter R, Nguyen M, Soriano F, Vasquez N, Weiss K, Welch B, Seubert P, Schenk D, Yednock T (2000). Peripherally administered antibodies against amyloid beta-peptide enter the central nervous system and reduce pathology in a mouse model of Alzheimer disease. Nat Med.

[B12] DeMattos RB, Bales KR, Cummins DJ, Dodart JC, Paul SM, Holtzman DM (2001). Peripheral anti-A beta antibody alters CNS and plasma A beta clearance and decreases brain A beta burden in a mouse model of Alzheimer's disease. Proc Natl Acad Sci USA.

[B13] Orgogozo JM, Gilman S, Dartigues JM, Laurent B, Puel M, Kirby LC, Jouanny P, Dubois B, Eisner L, Flitman S, Michel BF, Boada M, Frank A, Hock C (2003). Subacute meningoencephalitis in a subset of patients with AD after Abeta42 immunization. Neurology.

[B14] Masliah E, Hansen L, Adame A, Crews L, Bard F, Lee C, Seubert P, Games D, Kirby L, Schenk D (2005). Abeta vaccination effects on plaque pathology in the absence of encephalitis in Alzheimer disease. Neurology.

[B15] Nicoll JA, Wilkinson D, Holmes C, Steart P, Markham H, Weller RO (2003). Neuropathology of human Alzheimer disease after immunization with amyloid-beta peptide: a case report. Nat Med.

[B16] Ferrer I, Rovira MB, Guerra MLS, Rey MJ, Costa-Jussa F (2004). Neuropathology and pathogenesis of encephalitis following amyloid-beta immunization in Alzheimer's disease. Brain Pathol.

[B17] Furlan R, Brambilla E, Sanvito F, Roccatagliata L, Olivieri S, Bergami A, Pluchino S, Uccelli A, Comi G, Martino G (2003). Vaccination with amyloid-beta peptide induces autoimmune encephalomyelitis in C57/BL6 mice. Brain.

[B18] Pfeifer M, Boncristiano S, Bondolfi L, Stalder A, Deller T, Staufenbiel M, Mathews PM, Jucker M (2002). Cerebral hemorrhage after passive anti-Abeta immunotherapy. Science.

[B19] DeMattos RB, Boone LI, Hepburn DL, Parsadanian M, Bryan MT, Ness DK, Piroozi KS, Holtzman DM, Bales KR, Gitter BD, Paul SM, Racke M (2004). In vitro and in vivo characterization of beta-amyloid antibodies binding to cerebral amyloid angiopathy (CAA) and the selective exacerbation of CAA-associated microhemorrhage. Neurobiol Aging.

[B20] Wilcock DM, Rojiani A, Rosenthal A, Subbarao S, Freeman MJ, Gordon MN, Morgan D (2004). Passive immunotherapy against Abeta in aged APP-transgenic mice reverses cognitive deficits and depletes parenchymal amyloid deposits in spite of increased vascular amyloid and microhemorrhage. J Neuroinflammation.

[B21] Wilcock DM, Alamed J, Gottschall PE, Grimm J, Rosenthal A, Pons J, Ronan V, Symmonds K, Gordon MN, Morgan D (2006). Deglycosylated anti-amyloid-beta antibodies eliminate cognitive deficits and reduce parenchymal amyloid with minimal vascular consequences in aged amyloid precursor protein transgenic mice. J Neurosci.

[B22] Wilcock DM, Jantzen PT, Li Q, Morgan D, Gordon MN (2007). Amyloid-b vaccination, but not nitro-NSAID treatment, increases vascular amyloid and microhemorrhage while both reduce parenchymal amyloid. Neuroscience.

[B23] Turner MW (1996). Mannose-binding lectin: the pluripotent molecule of the innate immune system. Immunology Today.

[B24] Vasta GR, Quesenberry M, Ahmed H, O'Leary N (1999). C-type lectins and galectins mediate innate and adaptive immune functions: their roles in the complement activation pathway. Dev Comp Immunol.

[B25] Tenner AJ (1999). Membrane receptors for soluble defense collagens. Current Opinion in Immunology.

[B26] Engering AJ, Cella M, Fluitsma DM, Hoefsmit EC, Lanzavecchia A, Pieters J (1997). Mannose receptor mediated antigen uptake and presentation in human dendritic cells. Advances in Experimental Medicine and Biology.

[B27] Linehan SA, Martinez-Pomares L, Gordon S (2000). Mannose receptor and scavenger receptor: two macrophage pattern recognition receptors with diverse functions in tissue homeostasis and host defense. Adv Exp Med Biol.

[B28] Gröger M, Holnthoner W, Maurer D, Lechleitner S, Wolff K, Mayr BB, Lubitz W, Petzelbauer P (2000). Dermal microvascular endothelial cells express the 180-kDa macrophage mannose receptor in situ and in vitro. J Immunol.

[B29] Galea I, Palin K, Newman TA, Van Rooijen N, Perry VH, Boche D (2005). Mannose receptor expression specifically reveals perivascular macrophages in normal, injured, and diseased mouse brain. Glia.

[B30] Ghiran I, Barbashov SF, Klickstein LB, Tas SW, Jensenius JC, Nicholson-Weller A (2000). Complement receptor 1/CD35 is a receptor for mannan-binding lectin. Journal of Experimental Medicine.

[B31] Kozono Y, Abe R, Kozono H, Kelly RG, Azuma T, Holers VM (1998). Cross-linking CD21/CD35 or CD19 increases both B7-1 and B7-2 expression on murine splenic B cells. J Immunol.

[B32] Molina H, Holers VM, Li B, Fung Y, Mariathasan S, Goellner J, Strauss-Schoenberger J, Karr RW, Chaplin DD (1996). Markedly impaired humoral immune response in mice deficient in complement receptors 1 and 2. Proceedings of the National Academy of Sciences of the United States of America.

[B33] Okawa Y, Howard CR, Steward MW (1992). Production of anti-peptide specific antibody in mice following immunization with peptides conjugated to mannan. Journal of Immunological Methods.

[B34] Apostolopoulos V, Pietersz GA, Loveland BE, Sandrin MS, McKenzie IF (1995). Oxidative/reductive conjugation of mannan to antigen selects for T1 or T2 immune responses. Proceedings of the National Academy of Sciences of the United States of America.

[B35] Apostolopoulos V, Barnes N, Pietersz GA, McKenzie IFC (2000). Ex vivo targeting of the macrophage mannose receptor generates anti-tumor CTL responses. Vaccine.

[B36] Fearon DT, Locksley RM (1996). The instructive role of innate immunity in the acquired immune response. Science.

[B37] Apostolopoulos V, McKenzie IF (2001). Role of the mannose receptor in the immune response. Curr Mol Med.

[B38] Vaughan HA, Ho DW, Karanikas VA, Ong CS, Hwang LA, Pearson JM, McKenzie IF, Pietersz GA (1999). Induction of humoral and cellular responses in cynomolgus monkeys immunised with mannan-human MUC1 conjugates. Vaccine.

[B39] Ghochikyan A, Petrushina I, Lees A, Vasilevko V, Movsesyan N, Karapetyan A, Agadjanyan MG, Cribbs DH (2006). Ab-immunotherapy for Alzheimer's disease using mannan-amyloid-beta peptide immunoconjugates. DNA and Cell Biology.

[B40] Lees A, Nelson BL, Mond JJ (1996). Activation of soluble polysaccharides with 1-cyano-4-dimethylaminopyridinium tetrafluoroborate for use in protein-polysaccharide conjugate vaccines and immunological reagents. Vaccine.

[B41] Hsiao K, Chapman P, Nilsen S, Eckman C, Harigaya Y, Younkin S, Yang F, Cole G (1996). Correlative memory deficits, Aβ elevation, and amyloid plaques in transgenic mice. Science.

[B42] Frautschy SY F, Irrizarry M, Hyman B, Saido TC, Hsiao K, Cole GM (1998). Microglial response to amyloid plaques in APPsw transgenic mice. American Journal of Pathology.

[B43] Lesne S, Koh MT, Kotilinek L, Kayed R, Glabe CG, Yang A, Gallagher M, Ashe KH (2006). A specific amyloid-beta protein assembly in the brain impairs memory. Nature.

[B44] Cribbs DH, Ghochikyan A, Tran M, Vasilevko V, Petrushina I, Sadzikava N, Kesslak P, Kieber-Emmons T, Cotman CW, Agadjanyan MG (2003). Adjuvant-dependent modulation of Th1 and Th2 responses to immunization with beta-amyloid. Int Immunol.

[B45] Ghochikyan A, Vasilevko V, Petrushina I, Tran M, Sadzikava N, Babikyan D, Movsesyan N, Tian W, Ross TM, Cribbs DH, Agadjanyan MG (2003). Generation and chracterization of the humoral immune response to DNA immunization with a chimeric β-amyloid-interleukin-4 minigene. Eur J Immunol.

[B46] Petrushina I, Tran M, Sadzikava N, Ghochikyan A, Vasilevko V, Agadjanyan MG, Cribbs DH (2003). Importance of IgG2c isotype in the immune response to b-amyloid in APP/Tg mice. Neurosci Letters.

[B47] Mamikonyan G, Necula M, Mkrtichyan M, Ghochikyan A, Petrushina I, Movsesyan N, Mina E, Kiyatkin A, Glabe C, Cribbs DH, Agadjanyan MG (2007). Anti-Abeta 1–11 antibody binds to different beta-amyloid species, inhibits fibril formation, and disaggregates preformed fibrils, but not the most toxic oligomers. J Biol Chem.

[B48] Kayed R, Head E, Thompson JL, McIntire TM, Milton SC, Cotman CW, Glabe CG (2003). Common structure of soluble amyloid oligomers implies common mechanism of pathogenesis. Science.

[B49] Petrushina I, Ghochikyan A, Mktrichyan M, Mamikonyan G, Movsesyan N, Davtyan H, Patel A, Head E, Cribbs DH, Agadjanyan MG (2007). Alzheimer's Disease Peptide Epitope Vaccine Reduces Insoluble But Not Soluble/Oligomeric A{beta} Species in Amyloid Precursor Protein Transgenic Mice. J Neurosci.

[B50] Movsesyan N, Ghochikyan A, Mkrtichyan M, Petrushina I, Davtyan H, Olkhanud PB, Head E, Biragyn A, Cribbs DH, Agadjanyan MG (2008). Reducing AD-like pathology in 3xTg-AD mouse model by DNA epitope vaccine- a novel immunotherapeutic strategy. PLoS ONE.

[B51] Winkler DT, Bondolfi L, Herzig MC, Jann L, Calhoun ME, Wiederhold KH, Tolnay M, Staufenbiel M, Jucker M (2001). Spontaneous hemorrhagic stroke in a mouse model of cerebral amyloid angiopathy. J Neurosci.

[B52] Head E, Azizeh BY, Lott IT, Tenner AJ, Cotman CW, Cribbs DH (2001). Complement Association with Neurons and B-Amyloid Deposition in the Brains of Aged Individuals with Down Syndrome. Neurobiology of Disease.

[B53] Finkelman FD, Holmes J, Katona IM, Urban JF, Beckmann MP, Park LS, Schooley KA, Coffman RL, Mossmann TR, Paul WE (1990). Lymphokine control of in vivo immunoglobulin isotype selection. Annu Rev Immunol.

[B54] Chauhan NB, Siegel GJ (2005). Efficacy of anti-Abeta antibody isotypes used for intracerebroventricular immunization in TgCRND8. Neurosci Lett.

[B55] Morgan D, Gordon MN, Tan J, Wilcock D, Rojiani AM (2005). Dynamic complexity of the microglial activation response in transgenic models of amyloid deposition: implications for Alzheimer therapeutics. J Neuropathol Exp Neurol.

[B56] Wilcock DM, DiCarlo G, Henderson D, Jackson J, Clarke K, Ugen KE, Gordon MN, Morgan D (2003). Intracranially administered anti-Abeta antibodies reduce beta-amyloid deposition by mechanisms both independent of and associated with microglial activation. J Neurosci.

[B57] Wilcock DM, Munireddy SK, Rosenthal A, Ugen KE, Gordon MN, Morgan D (2004). Microglial activation facilitates Abeta plaque removal following intracranial anti-Abeta antibody administration. Neurobiol Dis.

[B58] Chung H, Brazil MI, Soe TT, Maxfield FR (1999). Uptake, degradation, and release of fibrillar and soluble forms of Alzheimer's amyloid beta-peptide by microglial cells. J Biol Chem.

[B59] Simard AR, Soulet D, Gowing G, Julien JP, Rivest S (2006). Bone marrow-derived microglia play a critical role in restricting senile plaque formation in Alzheimer's disease. Neuron.

[B60] Dodart JC, Bales KR, Gannon KS, Greene SJ, DeMattos RB, Mathis C, DeLong CA, Wu S, Wu X, Holtzman DM, Paul SM (2002). Immunization reverses memory deficits without reducing brain Abeta burden in Alzheimer's disease model. Nat Neurosci.

[B61] Frenkel D, Maron R, Burt DS, Weiner HL (2005). Nasal vaccination with a proteosome-based adjuvant and glatiramer acetate clears beta-amyloid in a mouse model of Alzheimer disease. J Clin Invest.

[B62] Schroeter S, Khan K, Barbour R, Doan M, Chen M, Guido T, Gill D, Basi G, Schenk D, Seubert P, Games D (2008). Immunotherapy reduces vascular amyloid-beta in PDAPP mice. J Neurosci.

[B63] Schenk D (2002). Opinion: Amyloid-beta immunotherapy for Alzheimer's disease: the end of the beginning. Nat Rev Neurosci.

[B64] Bacskai BJ, Kajdasz ST, McLellan ME, Games D, Seubert P, Schenk D, Hyman BT (2002). Non-Fc-mediated mechanisms are involved in clearance of amyloid-beta in vivo by immunotherapy. J Neurosci.

[B65] Kotilinek LA, Bacskai B, Westerman M, Kawarabayashi T, Younkin L, Hyman BT, Younkin S, Ashe KH (2002). Reversible memory loss in a mouse transgenic model of Alzheimer's disease. J Neurosci.

[B66] Gilman S, Koller M, Black RS, Jenkins L, Griffith SG, Fox NC, Eisner L, Kirby L, Rovira MB, Forette F, Orgogozo JM (2005). Clinical effects of Abeta immunization (AN1792) in patients with AD in an interrupted trial. Neurology.

[B67] Bayer AJ, Bullock R, Jones RW, Wilkinson D, Paterson KR, Jenkins L, Millais SB, Donoghue S (2005). Evaluation of the safety and immunogenicity of synthetic Abeta42 (AN1792) in patients with AD. Neurology.

[B68] Patton RL, Kalback WM, Esh CL, Kokjohn TA, Van Vickle GD, Luehrs DC, Kuo YM, Lopez J, Brune D, Ferrer I, Masliah E, Newel AJ, Beach TG, Castano EM, Roher AE (2006). Amyloid-beta peptide remnants in AN-1792-immunized Alzheimer's disease patients: a biochemical analysis. Am J Pathol.

[B69] Nicoll JA, Barton E, Boche D, Neal JW, Ferrer I, Thompson P, Vlachouli C, Wilkinson D, Bayer A, Games D, Seubert P, Schenk D, Holmes C (2006). Abeta species removal after abeta42 immunization. J Neuropathol Exp Neurol.

[B70] Engering AJ, Cella M, Fluitsma D, Brockhaus M, Hoefsmit EC, Lanzavecchia A, Pieters J (1997). The mannose receptor functions as a high capacity and broad specificity antigen receptor in human dendritic cells. Eur J Immunol.

[B71] Karanikas V, Hwang LA, Pearson J, Ong CS, Apostolopoulos V, Vaughan H, Xing PX, Jamieson G, Pietersz G, Tait B, Broadbent R, Thynne G, McKenzie IFC (1997). Antibody and T cell responses of patients with adenocarcinoma immunized with mannan-MUC1 fusion protein. Journal of Clinical Investigation.

[B72] Stambas J, Brown SA, Gutierrez A, Sealy R, Yue W, Jones B, Lockey TD, Zirkel A, Freiden P, Brown B, Surman S, Coleclough C, Slobod KS, Doherty PC, Hurwitz JL (2005). Long lived multi-isotype anti-HIV antibody responses following a prime-double boost immunization strategy. Vaccine.

[B73] Stambas J, Pietersz G, McKenzie I, Cheers C (2002). Oxidised mannan as a novel adjuvant inducing mucosal IgA production. Vaccine.

[B74] Stambas J, Pietersz G, McKenzie I, Nagabhushanam V, Cheers C (2002). Oxidised mannan-listeriolysin O conjugates induce Th1/Th2 cytokine responses after intranasal immunisation. Vaccine.

[B75] Vaughan HA, Ho DW, Karanikas V, Sandrin MS, McKenzie IF, Pietersz GA (2000). The immune response of mice and cynomolgus monkeys to macaque mucin 1-mannan. Vaccine.

[B76] Kawarabayashi T, Shoji M, Younkin LH, Wen-Lang L, Dickson DW, Murakami T, Matsubara E, Abe K, Ashe KH, Younkin SG (2004). Dimeric amyloid beta protein rapidly accumulates in lipid rafts followed by apolipoprotein E and phosphorylated tau accumulation in the Tg2576 mouse model of Alzheimer's disease. J Neurosci.

[B77] Das P, Chapoval S, Howard V, David CS, Golde TE (2003). Immune responses against Abeta1–42 in HLA class II transgenic mice: implications for Abeta1–42 immune-mediated therapies. Neurobiol Aging.

[B78] Lemere C, Spooner ET, LaFrancois J, Malester B, Mori C, Leverone JF, Matsuoka Y, Taylor JW, DeMattos RB, Holtzman DM, Clements JD, Selkoe DJ, Duff K (2003). Evidence for peripheral clearance of cerebral Abeta protein following chronic, active Abeta immunization in PSAPP mice. Neurobiol Dis.

[B79] Lemere CA, Spooner ET, Leverone JF, Mori C, Iglesias M, Bloom JK, Seabrook TJ (2003). Amyloid-beta immunization in Alzheimer's disease transgenic mouse models and wildtype mice. Neurochem Res.

[B80] Ellis RJ, Olichney JM, Thal LJ, Mirra SS, Morris JC, Beekly D, Heyman A (1996). Cerebral amyloid angiopathy in the brains of patients with Alzheimer's disease: the CERAD experience, Part XV. Neurology.

[B81] Tian J, Shi J, Mann DM (2004). Cerebral amyloid angiopathy and dementia. Panminerva Med.

[B82] Fryer JD, Simmons K, Parsadanian M, Bales KR, Paul SM, Sullivan PM, Holtzman DM (2005). Human apolipoprotein E4 alters the amyloid-beta 40:42 ratio and promotes the formation of cerebral amyloid angiopathy in an amyloid precursor protein transgenic model. J Neurosci.

[B83] Holtzman DM, Fagan AM, Mackey B, Tenkova T, Sartorius L, Paul SM, Bales K, Ashe KH, Irizarry MC, Hyman BT (2000). Apolipoprotein E facilitates neuritic and cerebrovascular plaque formation in an Alzheimer's disease model. Ann Neurol.

[B84] Racke MM, Boone LI, Hepburn DL, Parsadainian M, Bryan MT, Ness DK, Piroozi KS, Jordan WH, Brown DD, Hoffman WP, Holtzman DM, Bales KR, Gitter BD, May PC, Paul SM, DeMattos RB (2005). Exacerbation of cerebral amyloid angiopathy-associated microhemorrhage in amyloid precursor protein transgenic mice by immunotherapy is dependent on antibody recognition of deposited forms of amyloid beta. J Neurosci.

[B85] Lee EB, Leng LZ, Lee VM, Trojanowski JQ (2005). Meningoencephalitis associated with passive immunization of a transgenic murine model of Alzheimer's amyloidosis. FEBS Lett.

[B86] Miao J, Vitek MP, Xu F, Previti ML, Davis J, Van Nostrand WE (2005). Reducing cerebral microvascular amyloid-beta protein deposition diminishes regional neuroinflammation in vasculotropic mutant amyloid precursor protein transgenic mice. J Neurosci.

[B87] Herzig MC, Van Nostrand WE, Jucker M (2006). Mechanism of cerebral beta-amyloid angiopathy: murine and cellular models. Brain Pathol.

[B88] Fryer JD, Taylor JW, DeMattos RB, Bales KR, Paul SM, Parsadanian M, Holtzman DM (2003). Apolipoprotein E markedly facilitates age-dependent cerebral amyloid angiopathy and spontaneous hemorrhage in amyloid precursor protein transgenic mice. J Neurosci.

[B89] Eikelenboom P, Stam FC (1982). Immunoglobulins and complement factors in senile plaques. An immunoperoxidase study. Acta Neuropathol.

[B90] Eikelenboom P, Veerhuis R (1996). The role of complement and activated microglia in the pathogenesis of Alzheimer's disease. Neurobiol Aging.

